# Education of the Masses

**Published:** 1907-11

**Authors:** Geo. D. Young


### EDUCATION OF THE MASSES.

By Dr. Geo. D. Young.

Mr. President and members of the Florida State Dental Society: I have been led to the selection of this particular subject for a short paper by my own office experience and doubtless all of you have had the same experience and in consequence, will be somewhat interested in the discussion of this important subject. I say important, because the lack of knowledge of our profession tends to lessen the appreciation of the people for our services and consequently lessens the regard and respect for the profession as a true science. And it also appeals to us in a financial way, in that people will have that which they know is necessary for their comfort and health, and our services certainly are required for the enjoyment of either.

We all know that we could do much more for our patients if they only appreciated the necessity of our services.

There is no one subject practically speaking, about which people are more ignorant than about themselves and this is particularly true of that part of the anatomy which we call the mouth. There have been a number of ways suggested and tried of educating the people along these lines, some are good and should be continued while others are merely a waste of time and energy.

A few of the most prominent methods which have been suggested are:

(1). Instruction at the chair.

(2). To have a chapter in high school physiologies devoted exclusively to dentistry.

(3). To have a competent lecturer deliver a course of lectures to the school children.

(4). To have small booklets treating the subject thoroughly, for distribution among our patients.

Now let us take up these subjects separately and see what each offers to us.

I. Since Socrates went about Athens talking to all who would listen and in this simple way teaching them the philosophy of one of the greatest philosophers of all ages, namely his own—personal intercourse has been recognized as the best method of teaching and it is just as true between the dentist and his patients as between the philosopher and his pupil, but the present day dentist hasn't time to instruct his patient as he would like to, consequently we must look to some other method, however, we should do all we can at the chair.

II. The suggestion of the chapter in the physiologies cannot be used in this state as we would like, because we have no uniform text book law, so we will have to drop that idea for the present.

III. This third suggestion in a way takes the place of the second as we reach the school children, not through text books, but through lectures.

My idea would be to have a competent dentist in each town prepare and deliver before the school a short, concise course of lectures. I think we would have no trouble in getting the school boards' permission as they are progressive everywhere now and want to make the education of each pupil as complete as possible.

If a dentist in every town in the state would do this, it would certainly produce results of which the most sanguine of us have hardly dared to dream.

IV. I consider this last suggestion the most practical of all. The manufacturers of mouth washes have recognized the value of this plan in their advertising, but we can't afford to use their booklets because in so doing we would be lending our name to their advertisement and no professional man who does is worthy to be in a profession.

My idea would be to have a suitable booklet prepared by a committee from this Society and have it passed upon at our next meeting, then have a good supply printed and each member of this Society buy as many as he can distribute to advantage among his patients, have the indorsement of this Society printed upon each booklet so the people will have added confidence in it. There would be no objection to each dentist having his name and office address stamped upon his own booklets if he so desired.

Now as to the style of this booklet, there should be short, concise chapters upon the several different subjects of most importance, namely:

(1). Eruption of teeth.

(2). Importance of the teeth in the proper mastication of the food, also aesthetic appearance.

(3). Irregularities, their causes and means of correction.

(4). Care of the teeth—how to prevent decay.

(5). The saving of decayed teeth.

(6). Restoration of lost teeth by bridges and plates.

Each chapter to be supplemented, when necessary, by good charts and illustrations showing what can be done—there is nothing so convincing as a good illustration. It falls to the different professions to educate the people about their particular profession, and we as one of the most important and necessary professions must not shirk our duty—and we who constitute the dental profession in the State of Florida should strive to ever be at the front and never in the rear. I thank you.

